# The Genetic Architecture of Gliomagenesis–Genetic Risk Variants Linked to Specific Molecular Subtypes

**DOI:** 10.3390/cancers11122001

**Published:** 2019-12-12

**Authors:** Wendy Yi-Ying Wu, Gunnar Johansson, Carl Wibom, Thomas Brännström, Annika Malmström, Roger Henriksson, Irina Golovleva, Melissa L. Bondy, Ulrika Andersson, Anna M. Dahlin, Beatrice Melin

**Affiliations:** 1 Department of Radiation Sciences, Oncology, Umeå University, 901 87 Umeå, Sweden; wendy.wu@umu.se (W.Y.-Y.W.); gunnar.johansson@umu.se (G.J.); carl.wibom@umu.se (C.W.); roger.henriksson@umu.se (R.H.); ulrika.andersson@umu.se (U.A.); anna.dahlin@umu.se (A.M.D.); 2 Department of Medical Bioscience, Umeå University, 901 87 Umeå, Sweden; thomas.brannstrom@umu.se (T.B.); irina.golovleva@umu.se (I.G.); 3 Department of Advanced Home Care, Linköping University and Department of Clinical and Experimental Medicine, Linköping University, 581 83 Linköping, Sweden; annika.malmstrom@regionostergotland.se; 4 Department of Health Research and Policy (Epidemiology), Stanford University School of Medicine, Stanford, CA 94305, USA; mbondy@stanford.edu; 5 Department of Medicine, Dan L. Duncan Comprehensive Cancer Center, Baylor College of Medicine, Houston, TX 77030, USA

**Keywords:** glioma, *IDH* mutant, 1p/19q co-deletion, gliomagenesis, genotype phenotype, etiopathogenesis

## Abstract

Genome-wide association studies have identified 25 germline genetic loci that increase the risk of glioma. The somatic tumor molecular alterations, including *IDH*-mutation status and 1p/19q co-deletion, have been included into the WHO 2016 classification system for glioma. To investigate how the germline genetic risk variants correlate with the somatic molecular subtypes put forward by WHO, we performed a meta-analysis that combined findings from 330 Swedish cases and 876 controls with two other recent studies. In total, 5,103 cases and 10,915 controls were included. Three categories of associations were found. First, variants in *TERT* and *TP53* were associated with increased risk of all glioma subtypes. Second, variants in *CDKN2B-AS1*, *EGFR*, and *RTEL1* were associated with *IDH*-wildtype glioma. Third, variants in *CCDC26* (the 8q24 locus), *C2orf80* (close to *IDH*), *LRIG1*, *PHLDB1*, *ETFA*, *MAML2* and *ZBTB16* were associated with *IDH*-mutant glioma. We therefore propose three etiopathological pathways in gliomagenesis based on germline variants for future guidance of diagnosis and potential functional targets for therapies. Future prospective clinical trials of patients with suspicion of glioma diagnoses, using the genetic variants as biomarkers, are necessary to disentangle how strongly they can predict glioma diagnosis.

## 1. Introduction

Gliomas have several subtypes and the most recent update of glioma classification from the world health organization (WHO) 2016 added a layer of somatic molecular information to the classification of subtypes, including *IDH* mutation status and 1p/19q co-deletion [1]. The most aggressive type of glioma, glioblastoma (GBM), is defined as either *IDH*-wildtype (*IDH*-wt) or *IDH*-mutant. Primary GBM lacks *IDH* mutation and often has somatic promotor *TERT* mutations and *EGFR* amplification apart from high levels of mitosis and necrosis. Lower grade glioma (non-GBM) is classified morphologically into diffuse astrocytoma, oligodendroglioma, anaplastic astrocytoma, or anaplastic oligodendroglioma. This leads to three overarching molecular subgroups, namely *IDH*-wt, *IDH*-mutant and 1p/19q intact, or *IDH*-mutant and 1p/19q co-deleted [1].

Understanding the etiology and the development from a normal cell to a tumor cell is a powerful way to find methods for prevention and surveillance. It opens up the possibility to identify general mechanisms for tumor development that can be potential targets for treatment. Very few external etiological factors have been established for glioma apart from high doses of ionizing radiation, which is a rare exposure [2]. One possible assumption is that the etiology of glioma largely can be explained by genetic interplay between inherited genetic variants [3]. Not only high penetrant mutations in hereditary syndromes can lead to increased glioma risk, as recent genome-wide association studies identified 25 single nucleotide variants (SNVs) that were associated with glioma risk in adults [3,4,5,6,7,8]. Some of these germline genetic variants could potentially be used to support the tissue-based WHO pathological classification through blood diagnostics.

The aims of this study are to investigate how these genetic variants are distributed among the molecular sub-classifications of glioma used in WHO 2016 and to give an overview of which pathways could be targets for future functional studies of the genotypes and eventually drug development. We used 330 cases and 876 controls from the Swedish Glioma International Case-Control (GICC) study for our analyses. Furthermore, in order to provide robust results, we conducted a meta-analysis to combine our results with two recent large studies [9,10]. The genetic risk variants were then categorized into three groups, based on their pattern of association with glioma molecular subgroups: (1) SNVs in *TP53* and *TERT* associated with all glioma; (2) SNVs in *CDKN2B-AS1*, *EGFR*, near *EGFR*, and *RTEL1* associated with *IDH*-wt glioma; and (3) SNVs in *CCDC26*, *C2orf80*, *PHLDB1*, *ETFA*, *LRIG1*, *ZBTB16* and *MAML2* associated with *IDH*-mutant glioma.

## 2. Results

### 2.1. Descriptive Characteristics of Datasets

Table 1 shows the characteristics of 5103 cases and 10,915 controls in three studies. The ratios of control to case numbers were 2.65, 3.54, and 0.32 in the current, Labreche’s and Eckel-Passow’s studies, respectively [9,10]. The proportion of GBM cases was higher in our study (62.7%) than Eckel-Passow’s (41.9%) and Labreche’s studies (30.0%).

Labreche et al. and Eckel-Passow et al. classified cases into five molecular subgroups, based on *IDH* mutation, 1p/19q co-deletion and *TERT* promotor mutation [9,10]. The latter aberration is not formally a part of the WHO classification. For the purpose of the meta-analysis, we discarded the *TERT* promotor mutation status as a classifier, and regrouped all cases into three subtypes based on *IDH* mutation and 1p/19q co-deletion statuses; (1) *IDH*-wt subtype, including the previous groups “triple negative” and “*TERT* only”, (2) *IDH*-mutant and 1p/19q intact subtype, including the previous groups “*TERT-IDH*” and “*IDH*-only”, and (3) *IDH*-mutant and 1p/19q co-deleted subtype, including the previous group “triple positive”.

**Figure 1:**
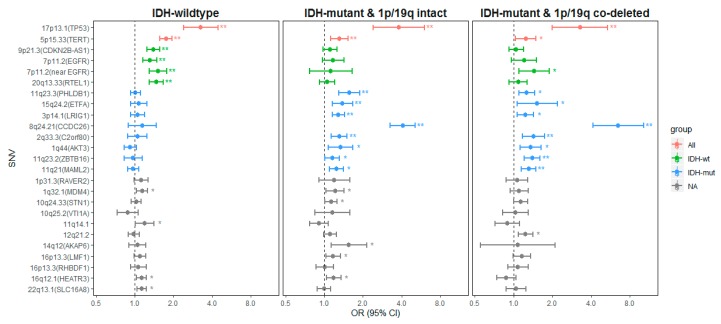
The associations between 25 germline variants and molecular subgroups in the meta-analysis. The genetic variants were categorized into three groups: (red) associated with all glioma (All), (green) associated with *IDH*-wildtype (*IDH*-wt), and (blue) associated with *IDH*-mutant (*IDH*-mut). * *p*-value < 0.05, ** Bonferroni *p*-value < 0.05/75. Abbreviations: SNV: single nucleotide variant; NA: Not significant for molecular subtypes in the present study; OR: odds ratio; CI: confidence interval.

**Figure 2:**
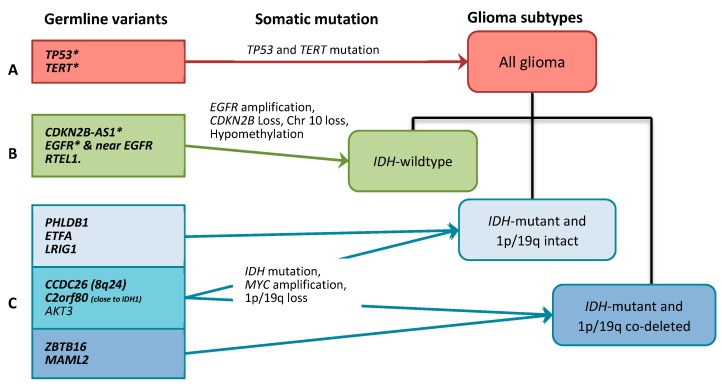
Three hypothesized pathways in gliomagenesis based on germline variants. (**A**) the overall glioma pathway, (**B**) the *IDH* wildtype pathway and (**C**) the *IDH* mutant pathway. The bold text represents statistically significant findings after adjusting for multiple comparisons. The asterisk (*) represents that the genes also have common somatic mutations in glioma.

The results of the meta-analysis and the OR (95% CI) in each study are shown in Figure 1, Figure S1 and Table S2. Based on our and previously published results, we suggest three different subtype-specific models of gliomagenesis as shown in Figure 2 and explained in detail below.

### 2.2. Gliomagenesis Models

#### 2.2.1. Genetic Variants Associated with all Glioma

According to the findings from the meta-analysis, two SNVs, rs78378222 (in the 3′ UTR region of *TP53*, 17p13.1) and rs10069690 (*TERT*, 5p15.33) were associated with increased risk of all glioma subtypes. Consistant and significant association between rs78378222 and all glioma types were found in all three studies (Figure S1). The ORs from the meta-analysis were 3.26 (95% CI = 2.40–4.43), 3.79 (95%CI = 2.40–6.00) and 3.28 (95%CI = 2.00–5.39), for *IDH*-wt, *IDH*-mutant and 1p/19q intact, and *IDH*-mutant and 1p/19q co-deleted subtypes, respectively (Table S2a). The rs10069690 had an association in the same direction for all glioma subtypes (OR = 1.75, 1.31 and 1.24 for *IDH*-wt, *IDH*-mutant and 1p/19q intact, and *IDH*-mutant and 1p/19q co-deleted, respectively), although *IDH*-mutant and 1p/19q co-deleted was not significant after adjusting for 25×3 tests (*p* = 0.018).

#### 2.2.2. Genetic Variants Associated with IDH-wt Glioma

Variants in 9p21.3 (*CDKN2B- AS1*), 7p11.2 (*EGFR*, intronic and near), and 20q13.33 (*RETL1*) (as indicated in green in Figure 1) were significantly associated with *IDH*-wt glioma. The ORs (95%CI) were 1.40 (1.25–1.57), 1.31 (1.15–1.48), 1.51 (1.29–1.77) and 1.47 (1.29–1.67), respectively (Table S2a). Variants in 1q32.1 (*MDM4*), 16q12.1 (*HEATR3*), 22q13.1 (*SLC16A8*) and 11q14.1 were also associated with increased risk of *IDH*-wt glioma although the results were not significant after adjusting for multiple comparison. Interestingly, the SNV rs75061357 at 7p11.2 (near *EGFR*) was also associated with *IDH*-mutant and 1p/19q co-deleted subtype (OR = 1.44, 95%CI = 1.09-1.89, *p*-value = 0.0092).

#### 2.2.3. Genetic Variants Associated with IDH-Mutant Glioma

Seven SNVs (indicated as blue in Figure 1) were statistically significantly associated with the *IDH*-mutant glioma subgroups. Strong associations were found between rs55705857 (*CCDC26*, 8q24.21) and *IDH* mutation (OR = 6.50 and 4.08 for *IDH*-mutant with and without 1p/19q co-deletion). The variant in *C2orf80* was associated with *IDH* mutation (OR = 1.42 and 1.31 for *IDH*-mutant with and without 1p/19q co-deletion). Risk variants in *LRIG1*, *PHLDB1*, *ETFA*, *MAML2* and *ZBTB16* were also associated with *IDH*-mutant glioma (Table S2a). After adjusting for multiple comparisons, we found that the *LRIG1*, *PHLDB1* and *ETFA* SNVs were significantly associated with *IDH*-mutant and 1p/19q intact subtype and the *MALM2* and *ZBTB16* SNVs were significantly associated with *IDH*-mutant and 1p/19q co-deleted subtype (Figure 1). One variant in 1q44 (*AKT3*) was also associated to the *IDH*-mutant subtypes regardless of 1p/19q status in all three studies, although the results from the meta-analysis were not statistically significant. We also observed that SNVs, rs4252707 (1p32.1, *MDM4*), rs11598018 (10q24.33, *STN1*), rs10131032 (14q12, *AKAP6*), rs3751667 (16p13.3, *LMF1*) and rs10852606 (16q12.1, *HEATR3*), were associated with the increased risk of *IDH*-mutant and 1p/19q intact subtype. The ORs (95%CI) were 1.22 (1.04–1.43), 1.13 (1.01–1.26), 1.55 (1.13–2.13), 1.18 (1.03–1.34) and 1.19 (1.04–1.35), respectively (Table S2a). However, none of them were significant after adjusting for 25×3 tests in the meta-analysis.

### 2.3. Genetic Effects on the Gene Expression Levels

To gain insight into the plausible mechanism of the germline variants, we conducted a search for the single-tissue expression quantitative trait loci (eQTL) and splicing expression quantitative trait loci (sQTL) among 25 glioma risk variants using data from 13 regions of normal brain from GTEx portal [11]. For SNVs associated with all subtypes, we didn’t find any significant differences on the gene expression levels (false discovery rate <0.05). Three SNVs associated with the *IDH*-wt subtype had influences on genes containing and/or neighboring the SNVs. Further, rs723527 (located in *EGFR*) was associated with expression of *EGFR*, rs634537 (located in *CDKN2B-AS1*) was associated with expression of *CDKN2A*, and rs2297440 (located in *RTEL1*) was associated with the expression of *RTEL1* and six other genes in the region. The variant, rs2297440, was also associated with alternative splicing in two genes, *RTEL1* and *LIME1* across several brain tissues. Among the SNVs associated with the *IDH*-mutant subtype, the risk allele (C) of rs11706832 was associated with higher *SLC25A26* gene expression levels in nucleus accumbens (basal ganglia) and substantia nigra. The risk variant, rs12803321 was associated with higher expression levels of *PHLDB1* and lower expression levels of *PR11-158I9.8*. The variant was also significantly associated with *PHLDB1* splicing. The results were summarized in the Table S3a–b.

## 3. Discussion

### 3.1. All Glioma Risk Genes

Defining the etiopathogenesis of different cancer types can be useful for several clinical applications. One example is to develop a biomarker panel for diagnostics of symptomatic patients as it has been successfully done in other diagnoses [12]. Increased basic knowledge of the mechanisms of glioma development could also help discover novel therapeutic targets, in analogy with how *PARP* inhibitors were discovered as a therapy in *BRCA1* positive breast cancer patients [13]. For glioma, there is no robust blood test in diagnostics, even if some different pre-diagnostic biomarkers such as metabolites have been suggested [14]. Current therapeutic options have limited success and novel effective targeted treatments for glioma are urgently needed to improve survival.

The present study highlights a clear pattern of germline genetic variants associated with *IDH*-mutant and *IDH*-wildtype glioma, respectively (Figure 2). The variants with lowest frequency and strongest effect size are those most likely to be possible to incorporate in a clinical setting as it was shown in the recent study by Eckel-Passow et al. [10]. In the meta-analyses, we observe an association with all glioma for the *TP53* gene variant and the *TERT* gene variant (Figure 1, group all, indicated in red). The *TP53* variant is relatively rare, and its function is associated with both somatic loss of heterozygosity in the region and disrupted *TP53* mRNA termination, as shown through previous studies of expression arrays in the TCGA dataset [15,16]. We did not find any differences in expression in the eQTL analyses (Table S3a). Furthermore, *TP53* mutations are generally rare in glioma families [17,18]. *TP53* is currently tested as a target for therapy, but as yet, no method has been introduced as a common treatment in any type of cancer [18].

*TERT* encodes for telomerase reverse transcriptase, which has a function in telomere maintenance [19]. The *TERT* genetic risk variant has been associated with both glioma risk and telomere length indicating a functional effect through telomere regulation [20,21]. Longer leukocyte telomere length has been associated with increased risk of glioma [22,23]. The direct analyses of the single variant in *TERT* did not show any association with gene expression. However, recent transcriptome-wide association analysis (TWAS) using gene-based approaches that aggregate the effects of multiple variants suggests that the effect of *TERT* gene variants is mediated by transcription levels of the *TERT* gene [24]. The TWAS reported an association between genetically predicted *TERT* gene expression levels and risk of both GBM and non-GBM, which is in line with our finding that the *TERT* gene variant is associated with risk of glioma, regardless of *IDH* mutation status. One plausible mechanism of action for the *TERT* genetic variant is through methylation changes, considering that variants in the genomic area have been associated with lower methylation of a CpG site near the *TERT* transcription start site, cg23827991 [25,26]. Furthermore, the risk allele of rs10069690 has been shown to create an alternative splice donor site leading to a decrease in telomerase activity [26,27]. The genomic area is pleiotropic, and it is also possible that the variant exerts its function through a telomerase-independent pathway (alternative lengthening of telomeres) rather than by upregulating telomerase activity [28]. Several clinical trials that target *TERT* and telomere function are ongoing for different types of diseases but currently not for glioma [19].

### 3.2. IDH-wildtype Glioma Risk Variants

For *IDH*-wt tumors, there was a strong consistent association with genetic variants in *EGFR*, *CDKN2B-AS1*, and the *RTEL* gene (Figure 1, group *IDH*-wt indicated in green). Interestingly, *EGFR* amplification and homozygous deletion of the chromosomal region comprising *CDKN2B-AS1* are two of the most common somatic mutations found in *IDH*-wt tumors, while the same aberrations are uncommon in *IDH*-mutant tumors [29]. This specifically highlights the importance of these two genes in the development of *IDH*-wt tumors. The understanding of the initiation of *EGFR* amplification and the functional aspects thereof are still under investigation [30]. One hypothesis of the link between genetic variants and the risk of subtype-specific disease could be that the genetic variants convey their increased glioma risk through contributing to a chronical activation of the *EGFR* receptor family that triggers tumor initiation. This is supported by the observation that glioma cases had higher concentrations of s*EGFR* and s*ERBB2* protein levels in prediagnostic serum samples from individuals that later developed GBM compared to matched controls [31,32]. In the brain tissue eQTL results, we found that risk variant of rs723527, located in intron 1, was associated with lower levels of *EGFR* expression. A similar finding was also found in the TWAS that increased expression of *EGFR* was negatively associated with glioma risk [24]. The mechanism behind how the genetic variant may be related to the development of aberrations in *EGFR* in GBM is currently unclear. The unexpected finding of lower expression linked to the *EGFR* variant could indicate different mechanisms in the initiation phase towards glioma development, compared with the fully developed glioblastoma stage [24].

In addition to the gain and amplification of *EGFR*, GBM has frequent somatic loss of chromosome 9p, which harbours the *CDKN2B-AS1* [33]. In previous studies, we have observed a subset of GBMs that have a specific *EGFR* germline gene variant in combination with somatic loss of *CDKN2B*, allelic loss of *EGFR*, and gain of the remaining *EGFR* allele [34]. This indicates that there may be subgroups of GBMs that have specific tumor features depending on their germline genetic variant status. Additional studies have shown that the *EGFR* glioma risk variants have been associated with *CDKN2B-AS1* loss and *EGFR* amplification [9,35]. We have sequenced a set of 14 glioma cases with this combination without finding any exonic mutations (unpublished observations). The lack of findings of germline mutations that are in linkage disequilibrium with the germline risk variants suggests other mechanisms of action, such as possibly enhancer or splicing effects. Families with glioma and melanoma have in rare cases been observed with germline mutations in *CDKN2A/B* region [17,36,37]. The result of tissue eQTL suggests a negative association between rs634537 and *CDKN2A* expression in brain cortex. The recent TWAS also reported an increased risk of GBM with lower *CDKN2B* expression levels [24]. Notable, the expression of *CDKN2B-AS1 (ANRIL), CDKN2A* and *CDKN2B* is correlated to each other [38]. Those findings were in line with the probable role of *CDKN2B* as a tumor suppressor gene. However, the exact functional mechanism of the germline genetic variants in the same region is not clear as an integrated analysis of TCGA dataset did not observe any clear link to chromosomal loss or mRNA expression levels of genes at the same genomic locus in the tumors [39].

*IDH*-wt tumors have lower levels of DNA-methylation compared to *IDH*-mutant tumors. In a recent study, we found that the methylation pattern of the tumor was associated with two genetic variants in *CDKN2B-AS1*, i.e., rs1412829 and rs4977756, and the same direction was seen in TCGA dataset [25]. The risk alleles of these variants were associated with lower levels of DNA methylation. This implicates that the functional role of genetic risk variants in the 9p21.3 region may not only be through direct regulation of the nearby tumor suppressor genes *CDKN2A/B*, but also through distant regulatory effects. Previous studies have shown that the combination of *CDKN2B* loss and *EGFR* amplification are early evolutionary events in GBM as they can be observed as a joint event in all parts of the tumor. Functional effects of the risk variants in the regulation of these genes are likely, even if the mechanisms of action still are to be further explored [40]. The mechanism of function in the genetic variant in *RTEL* is yet to be understood, and there are several other plausible genes in the chromosomal area that could be the actual gene with functional effect such as *STMN3* suggested by Labreche et al., or *DcR3,* a gene that has been differentially expressed in glioma and suggested as a therapeutic target by preclinical studies [41,42].

### 3.3. IDH-Mutant Glioma Risk Variants

As previously reported, the genetic variant at the 8p24 locus was strongly associated with *IDH*-mutant gliomas [9,10,43,44]. The variant maps to intron 1 of the long non-coding RNA *CCDC26* gene. It has been shown to have a superenhancer effect on the *MYC* locus (in close proximity to *CCDC26*) in hematopoietic malignancies [45]. A recent Turkish study of glioma cases showed some evidence of differential *MYC* expression depending on risk variant carriership indicating a regulation of the *MYC* locus [46]. There are currently ongoing studies with anti *MYC* compounds in animal models in conjunction with chemotherapy for other tumor types.

The genetic variant at *C2orf80* is located 46kb from the *IDH1* gene, which raises the question if it exerts a distance effect on *IDH1* itself. It was significantly associated with *IDH*-mutant glioma, irrespective of 1p/19q co-deletion. The function is not known, but *IDH1* is an interesting region as there are several *IDH* targeted therapies underway, currently tested both in acute myeloid leukemia and in glioma [47].

A non-significant but consistent association across the three studies between the variant in the *AKT3* gene and *IDH*-mutant subtype was observed. *AKT* has three different isoforms and those have been observed with differential expression in GBM mRNA levels [48]. *AKT3* mRNA was higher in lower grade gliomas and patients with tumors expressing higher levels of *AKT3* mRNA tended to have longer survival which has also been observed in patients with *IDH*-mutant subtype.

Furthermore, we observed a significant association between the genetic variants in *LRIG1*, *PHLDB1*, *ETFA*, *ZBTB16* and *MAML2* and the risk of *IDH*-mutant glioma. *LRIG1* regulates *EGFR*, and its soluble form has been shown to inhibit in vivo glioma growth irrespective of *EGFR* status. It exerts an effect as a tumor suppressor in glioma, possibly by a negative downregulation of the MET pathway but not AKT [49]. The *LRIG1* genetic risk variant is located in intron 2, relatively close to exon 3, where differential mRNA splicing is one potential functional mechanism, but the exact mechanism by which the increased glioma risk is conveyed is not understood. Functional studies of the *PHLDB1* gene with knockdown experiments in cell culture and 3D assays to evaluate the role of *PHLDB1* and *DDX6* suggest that both *PHLDB1* and *DDX6* may contribute to the cell viability [50]. TWAS has found an association between risk of non-GBM and the genetically predicted levels of *PHLDB1* expression, but also expression of other genes in the region, including *TREH*, *RPL5P30*, and *TMEM25* [24]. *ZBTB16* gene expression has been observed to have a distinct expression pattern associated with GBM prognosis, but no clear role has so far been observed for lower grade glioma tumors [51]. *MAML2* and *ETFA* are currently not established to have a clear role in somatic alterations in glioma. In summary, some genetic variants have suggested modes of function but many still need further investigation.

### 3.4. Limitations

Some associations were seen only in one or two studies and the mechanisms for this could be several, including differences in population substructures, the case ascertainment through the population based studies compared to tertiary referral centers for some studies and the strength of association and statistical power to detect associations. A prospective integrated study of all new patients having lesions with suspected glioma is necessary to determine the absolute effect of the glioma risk variants as a biomarker panel in the diagnostic setting.

## 4. Materials and Methods

### 4.1. Swedish GICC Study Population

The study subjects were those who participated in the Swedish arm of the Glioma International Case-Control (GICC) study. Details of patient recruitment, data collection and quality control were available in previous publications [5,52]. In brief, 18–80 years old cases were recruited between year 2010 and 2013 from five hospitals in Sweden. A total of 476 histologically confirmed newly diagnosed glioma cases and 924 population-based controls were included and genotyped. Subjects were excluded if their sample genotyping call-rate was <99% or reported sex and sex estimated by genotype were inconsistent. Those who were identified as outliers in principle component analyses, and one subject from each pair of subjects with spurious relations (PI-HAT > 0.2) were also excluded. After the quality control, 437 cases and 876 controls were included. A total of 330 patients with SNOMED codes 93,803, 93,823, 94,003, 94,013, 94,203, 94,403, 94,413, 94,503, and 94,513 were successfully obtained the unstained paraffin embedded slides for the classification of subtypes of glioma.

### 4.2. Genotyping and Imputation in Swedish GICC Study

Genotyping of germline DNA was performed using Illumnia’s Oncoaaray. Untyped variants were imputed using the IMPUTE2 software as previously described [5]. Imputation info scores for 25 SNVs are presented in Table S1. For imputed variants, genotypes were called based on the highest imputed genotype probability. A genotype call was set as “missing” in subjects where all three genotype probabilities for a variant were <0.9.

### 4.3. Immunohistochemistry in Swedish GICC Study

Based on eosin staining, a neuropathologist reviewed all slides to ensure that adequate tumor tissue was present on each slide. Slides were pretreated and stained against *IDH1-R132H* as previously described, using Ultra view DAB kit 760 (Roche, Basel Switzerland) and mouse anti *IDH1-R132H* (Dianova, Hamburg, Germany). All slides were coded to exclude any bias, and analyzed by the same neuropathologist. Representative staining for *IDH1-R132H* positive and negative staining were shown in Figure S2.

### 4.4. Fluorescent In Situ Hybridization (FISH) in Swedish GICC Study

Slides were first deparaffinized in xylene and dehydrated in 100% ethanol. Thereafter, slides were placed in a pretreatment solution for 12 min at 80 °C and protease solution for 45 min at 37 °C (according to the instruction in Vysis pretreatment kit IV (Abbot, Abbott Park, Illinois)). Then, slides were washed in H_2_O and dehydrated in a series of 70%, 80% and 100% ethanol. Sufficient amount of probes was added to cover each section. Slides were co-denatured at 80 °C for 6 min and hybridized over night at 37 °C. For 1p/19q co-deletion, we used “Vysis LSI 1p36/LSI 1q25 and LSI 19q13/19p13 dual-color probe” (Abbott). Unspecific staining was removed by using wash buffer 1 and 2 according to the instruction in the Vysis pretreatment kit IV (Abbott). Sections were counterstained using DAPI-Antifade (Cytocell, Cambridge, UK). Slides were coded to exclude any bias, and two independent observers analyzed 50 cells for each probe and analyzed as previously been described [35]. Representative staining for 1p/19q co-deletion was shown in Figure S3.

### 4.5. Statistical Analysis

We analyzed 25 SNVs which have been reported to show robustly significant association with the development of adult glioma in the recent GWAS study [5]. The associations between 25 SNVs and glioma subtypes were performed under an additive multinomial logistic regression model. We classified the molecular subtypes into three sub-groups including (1) *IDH*-wt (and 1p/19q intact), (2) *IDH*-mutant and 1p/19q intact and (3) *IDH*-mutant and 1p/19q co-deleted. Gender and first principle component (calculated using EIGENSOFT version 7.2.1) were used to adjust the potential confounding effects, such as population stratification [53,54]. To perform the meta-analysis, the OR and 95% CI of five molecular subtypes classified according to *IDH* mutation, 1p/19q co-deletion and *TERT* promotor mutation were first obtained. We then regrouped into three subtypes based on *IDH* mutation and 1p/19q co-deletion statuses as described above. A random-effects model was used to calculate summary ORs and 95% CIs using the R package “metafor” v2.1-0 [55].

### 4.6. Expression Quantitative Trait Locus Analysis

We used the V8 GTEx portal (https://gtexportal.org/home/) to investigate the association between 25 germline variants and gene expression levels on 13 regions of normal human brain tissue [11]. A false discovery rate of 0.05 was used to identify the gene with significant expression levels.

## 5. Conclusions

We used a meta-analysis approach to combine the results from two previously published studies and the present study, and observed a consistent and clear pattern of specific glioma risk variants associated with all glioma, *IDH*-wt and *IDH*-mutant glioma, respectively. Several of the 25 glioma risk variants map to, or are in close proximity to, genes that are frequently somatically changed in tumor tissue. Distant regulatory mechanisms or promoter functions have been suggested for some of variants, such as the variants mapping to 8p24, *MYC*, *TERT*, *PHDLB1*, and *TP53*. Further research into the molecular mechanisms behind the observed associations with increased glioma risk is necessary to improve the understanding of glioma etiopathogenesis. Moreover, it is conceivable that the risk variants eventually could be used as blood-based biomarkers, in conjunction with other pre-operative tools such as PET/MRI imaging, to determine the correct/suspected molecular classification before surgery. A presurgical diagnostic tool could open up for the possibility to give neoadjuvant treatment in a presurgical setting or to define the diagnosis and avoid surgery in vulnerable patients. That has to be determined by prospective trials testing a blood based biomarker test towards pre-surgery biopsies to see if the blood test can classify the molecular subtypes without the need of a surgical biopsy in symptomatic patients. The discovery of germline genetic risk variants has given novel insights into the etiology of glioma, but there are still several steps of systematic research needed before this knowledge may affect patient management.

## Figures and Tables

**Table 1:** Descriptive characteristics of datasets in three studies.

Study	Current Study	Labreche et al., 2018 [9]				Eckel-Passow et al., 2019 [10]		
		TCGA	French GWAS	French Sequencing	Subtotal Subjects	Mayo Clinic	UCSF	Subtotal Subjects
**Controls, N**	**876**	**2648**	**1190**	**5527**	**9365**	**443**	**231**	**674**
Male, N (%)	516 (58.9)	NA	NA	NA	NA	250 (56.4)	121 (54.4)	371 (55.0)
Age, median (range)	59 (21–82)	NA	NA	NA	NA	56 (22–84)	54 (18–89)	NA (18–89)
**Cases, N**	**330**	**521**	**1423**	**704**	**2648**	**1273**	**852**	**2125**
Male, N (%)	135 (40.9)	NA	NA	NA	NA	748 (58.8)	495 (48.1)	1243 (58.5)
Age, median (range)	59 (22–81)	NA	NA	NA	NA	48 (18–84)	51 (19–87)	NA (18–87)
GBM, N (%)	207 (62.7)	183 (35.12)	430 (30.2)	181 (25.7)	795 (30.0)	481 (37.8)	410 (48.1)	891 (41.9)
**Molecular subtypes**								
*IDH*-wildtype, N	258	55	450	277	782	165	335	500
*IDH*-mutant and 1p/19q intact, N	29	104	215	209	528	141	133	274
*IDH*-mutant and 1p/19q co-deleted, N	19	65	85	199	349	96	92	188

Abbreviations: NA: Not available, GBM: glioblastoma, GWAS: genome-wide association study.

## Supplementary Materials

The following are available online at https://www.mdpi.com/2072-6694/11/12/2001/s1, Figure S1: The forest plot of the meta-analysis by three molecular subtypes, Figure S2: Representative images showing IDH1-R132H positive/mutated (**a**) and negative/wildtype staining (**b**). Figure S3: Representative staining showing the quality of our 1p/19q co-deletion assay. Table S1: Overview of glioma risk single nucleotide variants at 25 loci, Table S2: (**a**) Summary odds ratio, 95% confidence interval and *p*-value for three molecular subtypes in the meta-analysis, (**b**) Odds ratio and 95% confidence interval for three molecular subtypes in current study, (**c**) Odds ratio and 95% confidence interval for three molecular subtypes in Eckel-Passow’s study, (**d**) Odds ratio and 95% confidence interval for three molecular subtypes in Labreche’s study. Table S3: (**a**) Results of single-tissue expression quantitative trait loci, (**b**) Results of single-tissue splicing expression quantitative trait loci.
