# Supplementary material for: The Genetic Architecture of Gliomagenesis–Genetic Risk Variants Linked to Specific Molecular Subtypes
Source: Cancers (Basel). 2019 Dec 12;11(12):2001. doi: 10.3390/cancers11122001 (PMC6966482; doi:10.3390/cancers11122001)

**Supplementary**

Table S1. Overview of glioma risk single nucleotide variants (SNVs) at the 25 loci

| SNV | Locus | Gene | Alleles* | RAF | Info |
|---|---|---|---|---|---|
| rs12752552 | 1p31.3 | RAVER2 | **T**/C | 0.88 | 0.9923 |
| rs4252707 | 1q32.1 | MDM4 | G/**A** | 0.18 | 0.9842 |
| rs12076373 | 1q44 | AKT3 | **G**/C | 0.82 | 0.9946 |
| rs7572263 | 2q33.3 | C2orf80 | **A**/G | 0.79 | 0.9848 |
| rs11706832 | 3p14.1 | LRIG1 | A/**C** | 0.45 | Genotyped |
| rs10069690 | 5p15.33 | TERT | C/**T** | 0.25 | Genotyped |
| rs75061358 | 7p11.2 | near EGFR | T/**G** | 0.08 | 0.9620 |
| rs723527 | 7p11.2 | EGFR | **A**/G | 0.54 | Genotyped |
| rs55705857 | 8q24.21 | CCDC26 | A/**G** | 0.08 | Genotyped |
| rs634537 | 9p21.3 | CDKN2B-AS1 | T/**G** | 0.48 | Genotyped |
| rs11598018 | 10q24.33 | STN1 | **C**/A | 0.47 | Genotyped |
| rs11599775 | 10q25.2 | VTI1A | **G**/A | 0.62 | 0.9817 |
| rs11233250 | 11q14.1 | NA | **C**/T | 0.88 | 0.9957 |
| rs7107785 | 11q21 | MAML2 | **T**/C | 0.49 | 0.9952 |
| rs648044 | 11q23.2 | ZBTB16 | **A**/G | 0.41 | Genotyped |
| rs12803321 | 11q23.3 | PHLDB1 | **G**/C | 0.68 | 0.9901 |
| rs1275600 | 12q21.2 | NA | **T**/A | 0.62 | 0.9957 |
| rs10131032 | 14q12 | AKAP6 | **G**/A | 0.91 | 0.9689 |
| rs77633900 | 15q24.2 | ETFA | G/**C** | 0.12 | Genotyped |
| rs2562152 | 16p13.3 | RHBDF1 | A/**T** | 0.89 | 0.9014 |
| rs3751667 | 16p13.3 | LMF1 | C/**T** | 0.21 | 0.9459 |
| rs10852606 | 16q12.1 | HEATR3 | T/**C** | 0.74 | 0.9880 |
| rs78378222 | 17p13.1 | TP53 | T/**G** | 0.02 | Genotyped |
| rs2297440 | 20q13.33 | RTEL1 | T/**C** | 0.79 | Genotyped |
| rs2235573 | 22q13.1 | SLC16A8 | **G**/A | 0.48 | 0.9935 |

Abbreviations: RAF: risk allele frequency; NA: unknown;

*The risk allele is emboldened.

Table S2. (a) Summary odds ratio (OR), 95% confidence interval (L95, U95) and p-value for three molecular subtypes in the meta-analysis. Overall p-value <0.05 is highlighted in bold and p-value<6.67E-04 (25×3 tests) is highlighted in red bold.

| | | | ***IDH*-wildtype** | | | | ***IDH*-mutant and 1p/19q intact** | | | | ***IDH*-mutant and 1p/19q co-deleted** | | | |
|---|---|---|---|---|---|---|---|---|---|---|---|---|---|---|
| SNV | Locus | Gene | OR | L95 | U95 | p-value | OR | L95 | U95 | p-value | OR | L95 | U95 | p-value |
| rs12752552 | 1p31.3 | RAVER2 | 1.12 | 0.99 | 1.27 | 6.94E-02 | 1.20 | 0.91 | 1.58 | 1.94E-01 | 1.06 | 0.88 | 1.28 | 5.34E-01 |
| rs4252707 | 1q32.1 | MDM4 | **1.14** | **1.03** | **1.26** | **9.27E-03** | **1.22** | **1.04** | **1.43** | **1.63E-02** | 1.10 | 0.94 | 1.30 | 2.29E-01 |
| rs12076373 | 1q44 | AKT3 | 0.92 | 0.83 | 1.03 | 1.43E-01 | **1.34** | **1.08** | **1.68** | **8.64E-03** | **1.36** | **1.12** | **1.64** | **1.85E-03** |
| rs7572263 | 2q33.3 | C2orf80 | 1.05 | 0.88 | 1.25 | 5.90E-01 | **1.31** | **1.14** | **1.51** | **1.97E-04** | **1.42** | **1.17** | **1.73** | **5.13E-04** |
| rs11706832 | 3p14.1 | LRIG1 | 1.06 | 0.93 | 1.20 | 4.06E-01 | **1.29** | **1.16** | **1.44** | **3.05E-06** | **1.23** | **1.06** | **1.43** | **5.74E-03** |
| rs10069690 | 5p15.33 | TERT | **1.75** | **1.57** | **1.96** | **6.26E-23** | **1.31** | **1.12** | **1.53** | **5.41E-04** | **1.24** | **1.04** | **1.48** | **1.80E-02** |
| rs75061358 | 7p11.2 | near EGFR | **1.51** | **1.29** | **1.77** | **2.87E-07** | 1.13 | 0.77 | 1.65 | 5.39E-01 | **1.44** | **1.09** | **1.89** | **9.20E-03** |
| rs723527 | 7p11.2 | EGFR | **1.31** | **1.15** | **1.48** | **2.58E-05** | 1.17 | 0.97 | 1.43 | 1.08E-01 | 1.20 | 0.96 | 1.51 | 1.03E-01 |
| rs55705857 | 8q24.21 | CCDC26 | 1.15 | 0.89 | 1.47 | 2.81E-01 | **4.08** | **3.26** | **5.10** | **4.27E-35** | **6.50** | **4.12** | **10.25** | **8.80E-16** |
| rs634537 | 9p21.3 | CDKN2B-AS1 | **1.40** | **1.25** | **1.57** | **5.77E-09** | 1.11 | 0.98 | 1.26 | 1.12E-01 | 1.05 | 0.92 | 1.19 | 4.85E-01 |
| rs11598018 | 10q24.33 | STN1 | 1.03 | 0.94 | 1.12 | 5.51E-01 | **1.13** | **1.01** | **1.26** | **2.75E-02** | 1.13 | 1.00 | 1.28 | 5.61E-02 |
| rs11599775 | 10q25.2 | VTI1A | 0.88 | 0.73 | 1.06 | 1.84E-01 | 1.16 | 0.85 | 1.59 | 3.51E-01 | 1.04 | 0.82 | 1.31 | 7.58E-01 |
| rs11233250 | 11q14.1 | NA | **1.20** | **1.01** | **1.42** | **3.74E-02** | 0.91 | 0.77 | 1.07 | 2.64E-01 | 0.89 | 0.71 | 1.11 | 2.93E-01 |
| rs7107785 | 11q21 | MAML2 | 0.97 | 0.88 | 1.08 | 5.93E-01 | **1.24** | **1.09** | **1.41** | **8.91E-04** | **1.31** | **1.15** | **1.48** | **3.39E-05** |
| rs648044 | 11q23.2 | ZBTB16 | 0.97 | 0.83 | 1.14 | 7.28E-01 | **1.15** | **1.01** | **1.32** | **3.60E-02** | **1.39** | **1.20** | **1.61** | **6.96E-06** |
| rs12803321 | 11q23.3 | PHLDB1 | 1.02 | 0.93 | 1.11 | 7.34E-01 | **1.57** | **1.30** | **1.88** | **1.88E-06** | **1.26** | **1.10** | **1.45** | **9.79E-04** |
| rs1275600 | 12q21.2 | NA | 0.98 | 0.89 | 1.08 | 7.09E-01 | 1.11 | 0.99 | 1.25 | 6.63E-02 | **1.24** | **1.08** | **1.41** | **1.53E-03** |
| rs10131032 | 14q12 | AKAP6 | 1.05 | 0.90 | 1.22 | 5.22E-01 | **1.55** | **1.13** | **2.13** | **6.90E-03** | 1.08 | 0.55 | 2.10 | 8.23E-01 |
| rs77633900 | 15q24.2 | ETFA | 1.07 | 0.93 | 1.24 | 3.46E-01 | **1.39** | **1.16** | **1.67** | **4.10E-04** | **1.52** | **1.06** | **2.18** | **2.23E-02** |
| rs2562152 | 16p13.3 | RHBDF1 | 1.07 | 0.93 | 1.23 | 3.32E-01 | 1.01 | 0.86 | 1.18 | 9.07E-01 | 1.08 | 0.90 | 1.30 | 4.07E-01 |
| rs3751667 | 16p13.3 | LMF1 | 1.10 | 0.99 | 1.22 | 7.29E-02 | **1.18** | **1.03** | **1.34** | **1.40E-02** | 1.16 | 0.99 | 1.35 | 5.82E-02 |

| | | | | | | | | | | | | | | |
|---|---|---|---|---|---|---|---|---|---|---|---|---|---|---|
| rs10852606 | 16q12.1 | HEATR3 | **1.13** | **1.03** | **1.24** | **9.07E-03** | **1.19** | **1.04** | **1.35** | **1.07E-02** | 0.88 | 0.74 | 1.05 | 1.50E-01 |
| rs78378222 | 17p13.1 | TP53 | **3.26** | **2.40** | **4.43** | **3.61E-14** | **3.79** | **2.40** | **6.00** | **1.20E-08** | **3.28** | **2.00** | **5.39** | **2.68E-06** |
| rs2297440 | 20q13.33 | RTEL1 | **1.47** | **1.29** | **1.67** | **2.47E-09** | 1.06 | 0.92 | 1.21 | 4.13E-01 | 1.08 | 0.92 | 1.27 | 3.26E-01 |
| rs2235573 | 22q13.1 | SLC16A8 | **1.13** | **1.04** | **1.23** | **2.54E-03** | 1.00 | 0.89 | 1.13 | 9.85E-01 | 1.04 | 0.87 | 1.25 | 6.41E-01 |

Abbreviations: **SNV**: single nucleotide variant

Table S2. (b) Odds ratio (OR) and 95% confidence interval (L95, U95) for three molecular subtypes in current study

| | | | ***IDH*-wildtype** | | | ***IDH*-mutant and 1p/19q intact** | | | ***IDH*-mutant and 1p/19q co-deleted** | | |
|---|---|---|---|---|---|---|---|---|---|---|---|
| SNV | Locus | Gene | OR | L95 | U95 | OR | L95 | U95 | OR | L95 | U95 |
| rs12752552 | 1p31.3 | RAVER2 | 1.21 | 0.87 | 1.69 | 2.55 | 0.78 | 8.29 | 0.90 | 0.34 | 2.38 |
| rs4252707 | 1q32.1 | MDM4 | 1.06 | 0.83 | 1.37 | 0.65 | 0.29 | 1.43 | 1.01 | 0.44 | 2.29 |
| rs12076373 | 1q44 | AKT3 | 0.95 | 0.74 | 1.23 | 1.06 | 0.53 | 2.11 | 1.84 | 0.65 | 5.23 |
| rs7572263 | 2q33.3 | C2orf80 | 0.93 | 0.73 | 1.17 | 2.69 | 1.06 | 6.83 | 1.49 | 0.62 | 3.59 |
| rs11706832 | 3p14.1 | LRIG1 | 1.21 | 0.99 | 1.47 | 0.99 | 0.59 | 1.68 | 1.67 | 0.87 | 3.20 |
| rs10069690 | 5p15.33 | TERT | 1.84 | 1.50 | 2.26 | 1.12 | 0.63 | 1.97 | 0.81 | 0.38 | 1.74 |
| rs75061358 | 7p11.2 | NA | 1.55 | 1.13 | 2.12 | 1.13 | 0.46 | 2.81 | 1.85 | 0.73 | 4.65 |
| rs723527 | 7p11.2 | EGFR | 1.38 | 1.13 | 1.69 | 1.25 | 0.73 | 2.13 | 1.15 | 0.60 | 2.22 |
| rs55705857 | 8q24.21 | CCDC26 | 0.98 | 0.66 | 1.44 | 2.97 | 1.43 | 6.16 | 4.43 | 1.90 | 10.36 |
| rs634537 | 9p21.3 | CDKN2B-AS1 | 1.45 | 1.19 | 1.77 | 1.02 | 0.61 | 1.72 | 1.34 | 0.71 | 2.55 |
| rs11598018 | 10q24.33 | STN1 | 1.03 | 0.84 | 1.25 | 1.15 | 0.68 | 1.97 | 1.96 | 1.00 | 3.86 |
| rs11599775 | 10q25.2 | VTI1A | 0.85 | 0.70 | 1.05 | 1.30 | 0.73 | 2.31 | 0.83 | 0.43 | 1.60 |
| rs11233250 | 11q14.1 | NA | 1.52 | 1.08 | 2.14 | 0.77 | 0.37 | 1.60 | 1.22 | 0.42 | 3.49 |
| rs7107785 | 11q21 | MAML2 | 1.03 | 0.85 | 1.25 | 2.01 | 1.15 | 3.53 | 1.01 | 0.54 | 1.90 |
| rs648044 | 11q23.2 | ZBTB16 | 0.85 | 0.69 | 1.04 | 1.36 | 0.80 | 2.31 | 1.46 | 0.77 | 2.79 |
| rs12803321 | 11q23.3 | PHLDB1 | 0.98 | 0.80 | 1.20 | 2.17 | 1.10 | 4.27 | 1.02 | 0.52 | 2.00 |
| rs1275600 | 12q21.2 | NA | 1.04 | 0.84 | 1.28 | 1.38 | 0.78 | 2.46 | 1.31 | 0.65 | 2.64 |
| rs10131032 | 14q12 | AKAP6 | 1.12 | 0.78 | 1.60 | 2.83 | 0.68 | 11.80 | 0.45 | 0.19 | 1.06 |
| rs77633900 | 15q24.2 | ETFA | 1.06 | 0.78 | 1.45 | 1.42 | 0.66 | 3.03 | 1.48 | 0.59 | 3.73 |
| rs2562152 | 16p13.3 | RHBDF1 | 1.12 | 0.80 | 1.57 | 2.02 | 0.63 | 6.45 | 0.99 | 0.35 | 2.77 |
| rs3751667 | 16p13.3 | LMF1 | 1.14 | 0.90 | 1.45 | 1.42 | 0.77 | 2.60 | 1.05 | 0.47 | 2.32 |
| rs10852606 | 16q12.1 | HEATR3 | 1.21 | 0.95 | 1.53 | 1.05 | 0.56 | 1.97 | 0.97 | 0.46 | 2.04 |
| rs78378222 | 17p13.1 | TP53 | 2.53 | 1.48 | 4.32 | 2.85 | 0.82 | 9.88 | 6.56 | 2.06 | 20.91 |
| rs2297440 | 20q13.33 | RTEL1 | 1.43 | 1.10 | 1.87 | 1.29 | 0.64 | 2.59 | 1.00 | 0.45 | 2.22 |

| | | | | | | | | | | |
|---|---|---|---|---|---|---|---|---|---|---|
| rs2235573 | 22q13.1 | SLC16A8 | 1.04 | 0.85 | 1.27 | 0.78 | 0.46 | 1.33 | 1.38 | 0.72 | 2.63 |

Abbreviations: **SNV**: single nucleotide variant

Table S2. (c) Odds ratio (OR) and 95% confidence interval (L95, U95) for three molecular subtypes in Eckel-Passow's study.

| | | | ***IDH*-wildtype** | | | ***IDH*-mutant and 1p/19q intact** | | | ***IDH*-mutant and 1p/19q co-deleted** | | |
|---|---|---|---|---|---|---|---|---|---|---|---|
| SNV | Locus | Gene | OR | L95 | U95 | OR | L95 | U95 | OR | L95 | U95 |
| rs12752552 | 1p31.3 | RAVER2 | 1.22 | 0.95 | 1.56 | 1.14 | 0.80 | 1.61 | 1.09 | 0.76 | 1.57 |
| rs4252707 | 1q32.1 | MDM4 | 1.15 | 0.95 | 1.39 | 1.14 | 0.88 | 1.47 | 1.04 | 0.78 | 1.40 |
| rs12076373 | 1q44 | AKT3 | 0.94 | 0.76 | 1.16 | 1.65 | 1.19 | 2.29 | 1.57 | 1.09 | 2.25 |
| rs7572263 | 2q33.3 | C2orf80 | 1.26 | 1.04 | 1.52 | 1.36 | 0.89 | 2.05 | 1.66 | 1.22 | 2.25 |
| rs11706832 | 3p14.1 | LRIG1 | 1.05 | 0.90 | 1.23 | 1.39 | 1.12 | 1.71 | 1.34 | 1.06 | 1.70 |
| rs10069690 | 5p15.33 | TERT | 1.84 | 1.54 | 2.20 | 1.48 | 1.17 | 1.88 | 1.14 | 0.86 | 1.50 |
| rs75061358 | 7p11.2 | NA | 1.25 | 0.55 | 2.85 | 0.93 | 0.45 | 1.94 | 1.72 | 1.15 | 2.57 |
| rs723527 | 7p11.2 | EGFR | 1.27 | 1.08 | 1.49 | 1.16 | 0.94 | 1.43 | 1.21 | 0.95 | 1.54 |
| rs55705857 | 8q24.21 | CCDC26 | 1.24 | 0.86 | 1.78 | 3.72 | 2.61 | 5.28 | 5.30 | 3.57 | 7.88 |
| rs634537 | 9p21.3 | CDKN2B-AS1 | 1.38 | 1.18 | 1.62 | 1.14 | 0.92 | 1.40 | 0.96 | 0.76 | 1.22 |
| rs11598018 | 10q24.33 | STN1 | 1.03 | 0.80 | 1.33 | 1.05 | 0.85 | 1.30 | 1.08 | 0.85 | 1.37 |
| rs11599775 | 10q25.2 | VTI1A | 1.03 | 0.65 | 1.64 | 1.11 | 0.76 | 1.61 | 1.07 | 0.84 | 1.37 |
| rs11233250 | 11q14.1 | NA | 1.21 | 0.93 | 1.57 | 0.81 | 0.59 | 1.11 | 0.74 | 0.52 | 1.05 |
| rs7107785 | 11q21 | MAML2 | 0.95 | 0.81 | 1.11 | 1.29 | 1.05 | 1.60 | 1.39 | 1.10 | 1.77 |
| rs648044 | 11q23.2 | ZBTB16 | 0.96 | 0.73 | 1.26 | 1.23 | 0.82 | 1.84 | 1.55 | 1.22 | 1.98 |
| rs12803321 | 11q23.3 | PHLDB1 | 0.96 | 0.81 | 1.12 | 1.47 | 1.17 | 1.86 | 1.15 | 0.89 | 1.48 |
| rs1275600 | 12q21.2 | NA | 0.88 | 0.75 | 1.04 | 1.21 | 0.97 | 1.52 | 1.20 | 0.93 | 1.54 |
| rs10131032 | 14q12 | AKAP6 | 0.97 | 0.73 | 1.29 | 1.58 | 1.02 | 2.44 | 1.63 | 0.99 | 2.67 |
| rs77633900 | 15q24.2 | ETFA | 1.20 | 0.90 | 1.58 | 1.52 | 1.07 | 2.15 | 1.97 | 1.37 | 2.85 |
| rs2562152 | 16p13.3 | RHBDF1 | 1.14 | 0.69 | 1.88 | 1.08 | 0.80 | 1.46 | 1.18 | 0.83 | 1.67 |
| rs3751667 | 16p13.3 | LMF1 | 1.16 | 0.96 | 1.40 | 1.03 | 0.80 | 1.33 | 1.13 | 0.85 | 1.51 |
| rs10852606 | 16q12.1 | HEATR3 | 1.13 | 0.95 | 1.35 | 1.16 | 0.79 | 1.69 | 0.99 | 0.77 | 1.28 |
| rs78378222 | 17p13.1 | TP53 | 3.17 | 1.72 | 5.86 | 5.27 | 2.55 | 10.90 | 3.17 | 1.33 | 7.51 |
| rs2297440 | 20q13.33 | RTEL1 | 1.66 | 1.34 | 2.05 | 1.05 | 0.80 | 1.37 | 1.08 | 0.80 | 1.46 |

| | | | | | | | | | | |
|---|---|---|---|---|---|---|---|---|---|---|
| rs2235573 | 22q13.1 | SLC16A8 | 1.13 | 0.97 | 1.33 | 1.12 | 0.91 | 1.39 | 0.90 | 0.71 | 1.14 |

Abbreviations: **SNV**: single nucleotide variant

Table S2. (d) Odds ratio (OR) and 95% confidence interval (L95, U95) for three molecular subtypes in Labreche's study.

| | | | ***IDH*-wildtype** | | | ***IDH*-mutant and 1p/19q intact** | | | ***IDH*-mutant and 1p/19q co-deleted** | | |
|---|---|---|---|---|---|---|---|---|---|---|---|
| SNV | Locus | Gene | OR | L95 | U95 | OR | L95 | U95 | OR | L95 | U95 |
| rs12752552 | 1p31.3 | RAVER2 | 1.07 | 0.91 | 1.25 | 1.17 | 0.73 | 1.90 | 1.06 | 0.84 | 1.33 |
| rs4252707 | 1q32.1 | MDM4 | 1.16 | 1.02 | 1.32 | 1.31 | 1.12 | 1.53 | 1.14 | 0.93 | 1.39 |
| rs12076373 | 1q44 | AKT3 | 0.91 | 0.78 | 1.05 | 1.25 | 1.03 | 1.51 | 1.26 | 1.00 | 1.58 |
| rs7572263 | 2q33.3 | C2orf80 | 0.99 | 0.87 | 1.12 | 1.28 | 1.10 | 1.49 | 1.31 | 1.09 | 1.57 |
| rs11706832 | 3p14.1 | LRIG1 | 0.95 | 0.80 | 1.12 | 1.28 | 1.12 | 1.45 | 1.15 | 0.98 | 1.34 |
| rs10069690 | 5p15.33 | TERT | 1.56 | 1.27 | 1.90 | 1.23 | 1.03 | 1.48 | 1.36 | 1.11 | 1.66 |
| rs75061358 | 7p11.2 | NA | 1.51 | 1.26 | 1.83 | 1.24 | 0.74 | 2.07 | 1.25 | 0.95 | 1.64 |
| rs723527* | 7p11.2 | EGFR | NA | NA | NA | NA | NA | NA | NA | NA | NA |
| rs55705857 | 8q24.21 | CCDC26 | 1.42 | 0.71 | 2.86 | 4.66 | 3.41 | 6.38 | 9.30 | 6.63 | 13.04 |
| rs634537 | 9p21.3 | CDKN2B-AS1 | 1.36 | 1.01 | 1.81 | 1.10 | 0.93 | 1.31 | 1.07 | 0.91 | 1.25 |
| rs11598018 | 10q24.33 | STN1 | 1.03 | 0.92 | 1.14 | 1.15 | 1.02 | 1.31 | 1.12 | 0.96 | 1.30 |
| rs11599775* | 10q25.2 | VTI1A | NA | NA | NA | NA | NA | NA | NA | NA | NA |
| rs11233250 | 11q14.1 | NA | 1.09 | 0.92 | 1.28 | 0.97 | 0.79 | 1.19 | 0.96 | 0.76 | 1.22 |
| rs7107785 | 11q21 | MAML2 | 0.95 | 0.80 | 1.14 | 1.17 | 1.03 | 1.34 | 1.29 | 1.11 | 1.50 |
| rs648044 | 11q23.2 | ZBTB16 | 1.09 | 0.93 | 1.27 | 1.13 | 0.98 | 1.31 | 1.31 | 1.11 | 1.55 |
| rs12803321 | 11q23.3 | PHLDB1 | 1.06 | 0.94 | 1.19 | 1.64 | 1.17 | 2.31 | 1.34 | 1.13 | 1.59 |
| rs1275600 | 12q21.2 | NA | 1.02 | 0.91 | 1.14 | 1.07 | 0.93 | 1.22 | 1.25 | 1.07 | 1.47 |
| rs10131032 | 14q12 | AKAP6 | 1.07 | 0.87 | 1.31 | 1.41 | 0.86 | 2.32 | 1.31 | 0.95 | 1.81 |
| rs77633900 | 15q24.2 | ETFA | 1.02 | 0.83 | 1.25 | 1.34 | 1.07 | 1.67 | 1.24 | 0.94 | 1.63 |
| rs2562152 | 16p13.3 | RHBDF1 | 1.05 | 0.90 | 1.23 | 0.97 | 0.80 | 1.17 | 1.05 | 0.84 | 1.31 |
| rs3751667 | 16p13.3 | LMF1 | 1.05 | 0.91 | 1.22 | 1.22 | 1.04 | 1.42 | 1.18 | 0.98 | 1.43 |
| rs10852606 | 16q12.1 | HEATR3 | 1.11 | 0.99 | 1.25 | 1.20 | 1.04 | 1.38 | 0.81 | 0.69 | 0.96 |
| rs78378222 | 17p13.1 | TP53 | 4.04 | 2.53 | 6.45 | 3.12 | 1.59 | 6.10 | 2.59 | 1.27 | 5.28 |
| rs2297440 | 20q13.33 | RTEL1 | 1.37 | 1.14 | 1.63 | 1.05 | 0.89 | 1.24 | 1.09 | 0.90 | 1.33 |

| | | | | | | | | | | |
|---|---|---|---|---|---|---|---|---|---|---|
| rs2235573 | 22q13.1 | SLC16A8 | 1.16 | 1.04 | 1.30 | 0.97 | 0.85 | 1.10 | 1.11 | 0.95 | 1.30 |

Abbreviations: **SNV**: single nucleotide variant

*Two SNPs (rs11979158 in EGFR and rs11196067 in VTI1A) were not included since the allele frequencies of the SNPs were different compared to the SNPs used in current and Eckel-Passow’s studies.

Table S3. (a) Results of single-tissue expression quantitative trait loci (eQTL) (Data obtained from GTEx V8 portal, https://gtexportal.org/home/, GTEx Analysis Release V8 (dbGaP Accession phs000424.v8.p2))

| SNV | Locus | Expression gene symbol | P-value | NES | Tissue |
|---|---|---|---|---|---|
| rs12752552 | 1p31.3 | JAK1 | 9.40E-11 | -0.55 | Brain - Cerebellum |
| | | JAK1 | 8.20E-10 | -0.47 | Brain - Cerebellar Hemisphere |
| | | JAK1 | 3.50E-06 | -0.35 | Brain - Cortex |
| | | JAK1 | 4.50E-05 | -0.3 | Brain - Frontal Cortex (BA9) |
| rs4252707 | 1q32.1 | | | | |
| rs12076373 | 1q44 | | | | |
| rs7572263 | 2q33.3 | | | | |
| rs11706832 | 3p14.1 | SLC25A26 | 2.60E-05 | 0.28 | Brain - Nucleus accumbens (basal ganglia) |
| | | SLC25A26 | 1.60E-05 | 0.42 | Brain - Substantia nigra |
| rs10069690 | 5p15.33 | | | | |
| rs75061358 | 7p11.2 | | | | |
| rs723527 | 7p11.2 | EGFR | 2.60E-05 | 0.18 | Brain - Cortex |
| rs55705857 | 8q24.21 | | | | |
| rs634537 | 9p21.3 | CDKN2A | 4.70E-05 | -0.33 | Brain - Cortex |
| rs11598018 | 10q24.33 | RP11-541N10.3 | 5.70E-10 | 0.44 | Brain - Cerebellum |
| | | RP11-541N10.3 | 9.60E-06 | 0.34 | Brain - Cortex |
| | | RP11-541N10.3 | 8.80E-07 | 0.33 | Brain - Frontal Cortex (BA9) |
| rs11599775 | 10q25.2 | | | | |
| rs11233250 | 11q14.1 | MIR4300HG | 6.80E-06 | 0.81 | Brain - Amygdala |
| | | MIR4300HG | 1.30E-06 | 0.7 | Brain - Anterior cingulate cortex (BA24) |
| | | MIR4300HG | 8.20E-06 | 0.45 | Brain - Caudate (basal ganglia) |
| | | MIR4300HG | 1.50E-05 | 0.64 | Brain - Cortex |
| | | MIR4300HG | 4.20E-10 | 0.7 | Brain - Hippocampus |
| | | MIR4300HG | 8.40E-07 | 0.65 | Brain - Hypothalamus |
| | | MIR4300H | 3.10E-07 | 0.76 | Brain - Spinal cord (cervical c-1) |

| | | | | | |
|---|---|---|---|---|---|
| | | G | | | |
| | | MIR4300HG | 5.70E-06 | 0.9 | Brain - Substantia nigra |
| rs7107785 | 11q21 | | | | |
| rs648044 | 11q23.2 | | | | |
| rs12803321 | 11q23.3 | PHLDB1 | 5.40E-08 | -0.36 | Brain - Cerebellar Hemisphere |
| | | PHLDB1 | 1.60E-08 | -0.33 | Brain - Cerebellum |
| | | RP11-158I9.8 | 1.80E-05 | 0.31 | Brain - Cerebellum |
| | | RP11-158I9.8 | 1.90E-05 | 0.3 | Brain - Hippocampus |
| | | RP11-158I9.8 | 8.40E-06 | 0.29 | Brain - Putamen (basal ganglia) |
| | | RP11-158I9.8 | 6.90E-06 | 0.36 | Brain - Spinal cord (cervical c-1) |
| rs1275600 | 12q21.2 | | | | |
| rs10131032 | 14q12 | | | | |
| rs77633900 | 15q24.2 | | | | |
| rs2562152 | 16p13.3 | IL9RP3 | 3.90E-06 | -0.74 | Brain - Cerebellar Hemisphere |
| | | RHBDF1 | 4.90E-05 | -0.47 | Brain - Cerebellar Hemisphere |
| rs3751667 | 16p13.3 | LMF1 | 7.10E-06 | 0.35 | Brain - Anterior cingulate cortex (BA24) |
| | | RP11-161M6.2 | 6.00E-07 | -0.27 | Brain - Caudate (basal ganglia) |
| | | LMF1 | 2.00E-05 | 0.32 | Brain - Caudate (basal ganglia) |
| | | LMF1 | 4.60E-07 | -0.38 | Brain - Cerebellar Hemisphere |
| | | LA16c-306A4.2 | 2.70E-06 | -0.37 | Brain - Cerebellar Hemisphere |
| | | LMF1 | 6.60E-06 | -0.34 | Brain - Cerebellum |
| | | RP11-161M6.2 | 4.40E-10 | -0.48 | Brain - Cortex |
| | | LMF1 | 9.30E-10 | 0.44 | Brain - Cortex |
| | | LMF1 | 9.80E-07 | 0.32 | Brain - Hippocampus |
| | | LMF1 | 2.40E-06 | 0.26 | Brain - Hypothalamus |
| | | LMF1 | 1.30E-08 | 0.32 | Brain - Nucleus accumbens (basal ganglia) |
| rs10852606 | 16q12.1 | HEATR3 | 4.90E-11 | 0.65 | Brain - Amygdala |
| | | HEATR3 | 3.00E-18 | 0.85 | Brain - Anterior cingulate cortex (BA24) |
| | | HEATR3 | 4.40E-23 | 0.67 | Brain - Caudate (basal ganglia) |
| | | HEATR3 | 1.40E-39 | 1 | Brain - Cerebellar Hemisphere |

| | | | | | |
|---|---|---|---|---|---|
| | | RP11-429P3.8 | 1.10E-08 | 0.4 | Brain - Cerebellar Hemisphere |
| | | HEATR3 | 6.80E-61 | 1.1 | Brain - Cerebellum |
| | | RP11-429P3.8 | 4.40E-16 | 0.66 | Brain - Cerebellum |
| | | HEATR3 | 2.50E-44 | 0.97 | Brain - Cortex |
| | | HEATR3 | 8.20E-23 | 0.75 | Brain - Frontal Cortex (BA9) |
| | | HEATR3 | 7.80E-20 | 0.8 | Brain - Hippocampus |
| | | HEATR3 | 1.60E-16 | 0.83 | Brain - Hypothalamus |
| | | HEATR3 | 2.20E-27 | 0.61 | Brain - Nucleus accumbens (basal ganglia) |
| | | HEATR3 | 2.80E-21 | 0.65 | Brain - Putamen (basal ganglia) |
| | | RP11-429P3.8 | 2.60E-05 | 0.4 | Brain - Putamen (basal ganglia) |
| | | HEATR3 | 1.00E-25 | 0.96 | Brain - Spinal cord (cervical c-1) |
| | | HEATR3 | 2.10E-14 | 0.68 | Brain - Substantia nigra |
| rs78378222 | 17p13.1 | | | | |
| rs2297440 | 20q13.33 | STMN3 | 6.90E-09 | -0.21 | Brain - Caudate (basal ganglia) |
| | | LIME1 | 2.00E-05 | -0.24 | Brain - Caudate (basal ganglia) |
| | | STMN3 | 5.20E-13 | -0.37 | Brain - Cerebellar Hemisphere |
| | | GMEB2 | 1.40E-11 | -0.52 | Brain - Cerebellar Hemisphere |
| | | ZGPAT | 1.60E-05 | 0.2 | Brain - Cerebellar Hemisphere |
| | | SLC2A4RG | 4.20E-05 | 0.22 | Brain - Cerebellar Hemisphere |
| | | STMN3 | 8.90E-11 | -0.34 | Brain - Cerebellum |
| | | SLC2A4RG | 2.40E-06 | 0.29 | Brain - Cerebellum |
| | | GMEB2 | 3.30E-06 | -0.36 | Brain - Cerebellum |
| | | STMN3 | 2.00E-10 | -0.21 | Brain - Cortex |
| | | RTEL1 | 1.00E-05 | 0.18 | Brain - Cortex |
| | | ARFRP1 | 2.60E-05 | 0.18 | Brain - Cortex |
| | | STMN3 | 1.80E-05 | -0.19 | Brain - Nucleus accumbens (basal ganglia) |
| | | STMN3 | 3.20E-07 | -0.23 | Brain - Putamen (basal ganglia) |
| | | LIME1 | 1.50E-05 | -0.28 | Brain - Putamen (basal ganglia) |
| | | ARFRP1 | 2.70E-05 | 0.22 | Brain - Putamen (basal ganglia) |
| rs2235573 | 22q13.1 | SLC16A8 | 1.10E-07 | -0.42 | Brain - Amygdala |
| | | PICK1 | 2.40E-06 | -0.2 | Brain - Caudate (basal ganglia) |
| | | SLC16A8 | 5.20E-05 | -0.19 | Brain - Cortex |
| | | PICK1 | 7.40E-09 | -0.26 | Brain - Hippocampus |
| | | SLC16A8 | 3.10E-06 | -0.25 | Brain - Hippocampus |
| | | CTA-228A9.3 | 9.30E-06 | -0.2 | Brain - Hippocampus |

| | | | |
|---|---|---|---|
| PICK1 | 4.10E-10 | -0.33 | Brain - Hypothalamus |
| PICK1 | 5.40E-10 | -0.32 | Brain - Putamen (basal ganglia) |
| PICK1 | 8.70E-14 | -0.6 | Brain - Spinal cord (cervical c-1) |
| PICK1 | 4.70E-06 | -0.29 | Brain - Substantia nigra |

Abbreviations: **NES:** Normalized effect size

Table S3. (b) Results of single-tissue splicing expression quantitative trait loci (sQTL) (Data obtained from GTEx V8 portal, https://gtexportal.org/home/, GTEx Analysis Release V8 (dbGaP Accession phs000424.v8.p2))

| SNV | Locus | Expression gene symbol | Intron id | P-value | NES | Tissue |
|---|---|---|---|---|---|---|
| rs12752552 | 1p31.3 | | | | | |
| rs4252707 | 1q32.1 | | | | | |
| rs12076373 | 1q44 | | | | | |
| rs7572263 | 2q33.3 | | | | | |
| rs11706832 | 3p14.1 | | | | | |
| rs10069690 | 5p15.33 | | | | | |
| rs75061358 | 7p11.2 | | | | | |
| rs723527 | 7p11.2 | | | | | |
| rs55705857 | 8q24.21 | | | | | |
| rs634537 | 9p21.3 | | | | | |
| rs11598018 | 10q24.33 | | | | | |
| rs11599775 | 10q25.2 | | | | | |
| rs11233250 | 11q14.1 | | | | | |
| rs7107785 | 11q21 | | | | | |
| rs648044 | 11q23.2 | | | | | |
| rs12803321 | 11q23.3 | PHLDB1 | 118607699:118613816:clu_7290 | 2.90E-10 | -0.66 | Brain - Caudate (basal ganglia) |
| | | PHLDB1 | 118613450:118613816:clu_6649 | 1.10E-12 | 0.77 | Brain - Hippocampus |
| | | PHLDB1 | 118607699:118613816:clu_7151 | 3.80E-09 | -0.64 | Brain - Hypothalamus |
| rs1275600 | 12q21.2 | | | | | |
| rs10131032 | 14q12 | | | | | |
| rs77633900 | 15q24.2 | | | | | |
| rs2562152 | 16p13.3 | | | | | |
| rs3751667 | 16p13.3 | LMF1 | 979758:981145:clu_13140 | 8.80E-18 | 0.78 | Brain - Nucleus accumbens (basal ganglia) |
| | | RP11-161M6.2 | 979758:981145:clu_13140 | 8.80E-18 | 0.78 | Brain - Nucleus accumbens (basal ganglia) |

| | | | | | | |
|---|---|---|---|---|---|---|
| rs10852606 | 16q12.1 | HEATR3 | 50066269:50066367:clu_15456 | 6.70E-13 | -0.71 | Brain - Cerebellum |
| | | HEATR3 | 50066269:50066367:clu_15456 | 6.70E-13 | -0.71 | Brain - Cerebellum |
| rs78378222 | 17p13.1 | | | | | |
| rs2297440 | 20q13.33 | RTEL1 | 63689132:63689750:clu_20190 | 2.00E-17 | -1.2 | Brain - Amygdala |
| | | RTEL1 | 63689132:63689750:clu_21705 | 2.60E-23 | -1.3 | Brain - Anterior cingulate cortex (BA24) |
| | | RTEL1 | 63689132:63689750:clu_24457 | 7.70E-30 | -1.2 | Brain - Caudate (basal ganglia) |
| | | RTEL1 | 63689132:63689750:clu_25892 | 1.50E-61 | -1.5 | Brain - Cerebellar Hemisphere |
| | | RTEL1 | 63689132:63689750:clu_25892 | 1.50E-61 | -1.5 | Brain - Cerebellar Hemisphere |
| | | RTEL1 | 63689132:63689750:clu_27064 | 1.20E-74 | -1.4 | Brain - Cerebellum |
| | | LIME1 | 63737902:63738008:clu_27080 | 1.6E-06 | 0.55 | Brain - Cerebellum |
| | | RTEL1 | 63689132:63689750:clu_25253 | 7.10E-48 | -1.2 | Brain - Cortex |
| | | RTEL1 | 63689132:63689750:clu_23443 | 3.90E-37 | -1.3 | Brain - Frontal Cortex (BA9) |
| | | RTEL1 | 63689132:63689750:clu_22333 | 4.60E-24 | -1.2 | Brain - Hippocampus |
| | | RTEL1 | 63689132:63689750:clu_23904 | 4.60E-29 | -1.1 | Brain - Hypothalamus |
| | | RTEL1 | 63689132:63689750:clu_24978 | 1.30E-41 | -1.3 | Brain - Nucleus accumbens (basal ganglia) |
| | | LIME1 | 63736186:63737533:clu_24993 | 5.20E-08 | 0.54 | Brain - Nucleus accumbens (basal ganglia) |

| | | | | | | |
|---|---|---|---|---|---|---|
| | | RTEL1 | 63689132:63689750:clu_21995 | 3.80E-34 | -1.3 | Brain - Putamen (basal ganglia) |
| rs2235573 | 22q13.1 | | | | | |

Abbreviations: **SNV:** single nucleotides variant; **NES:** Normalized effect size

Figure S1. The forest plot of the meta-analysis by three molecular subtypes.

L: Labreche et al. , E: Eckel-Passow et al. , M:current study

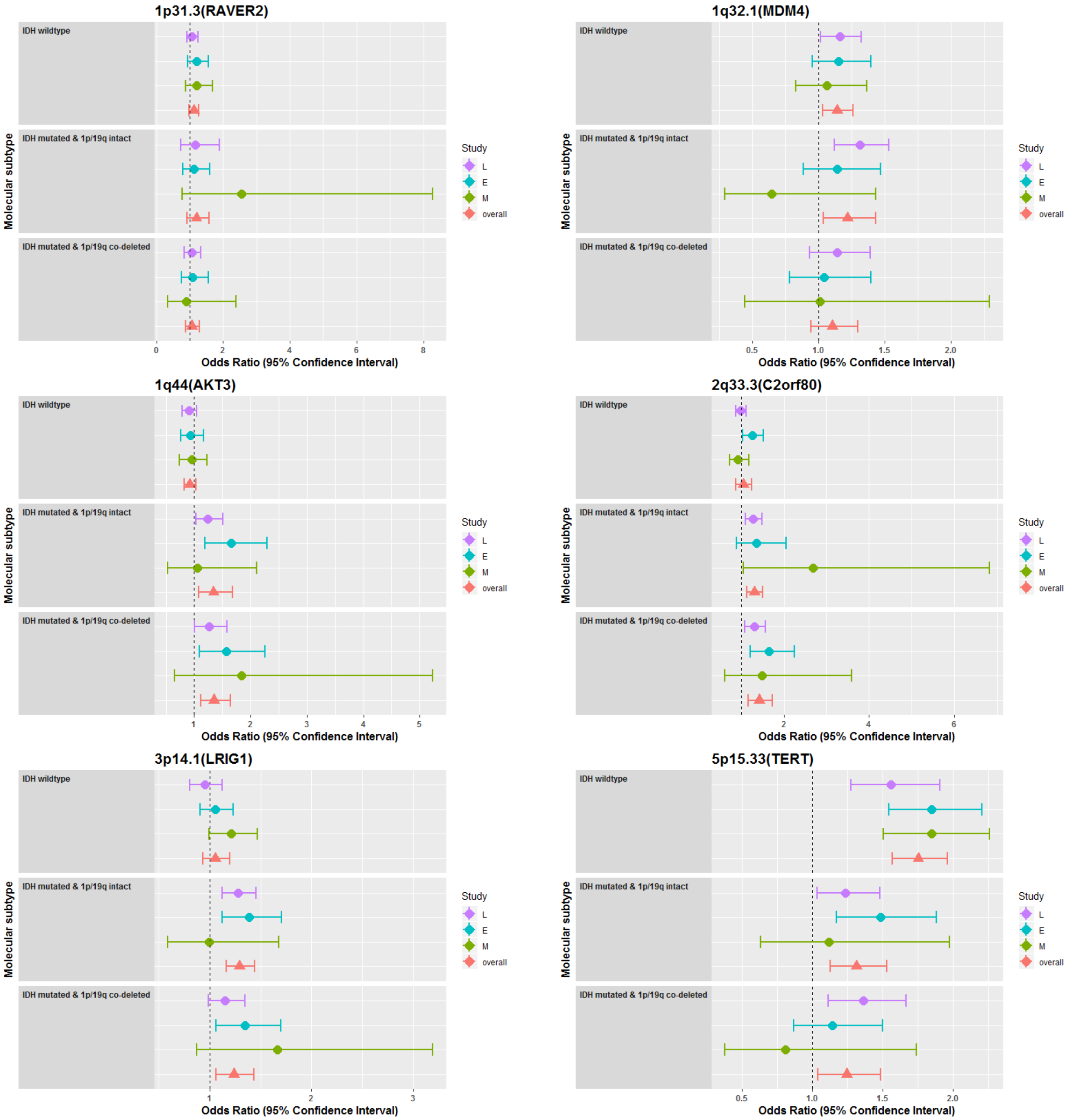

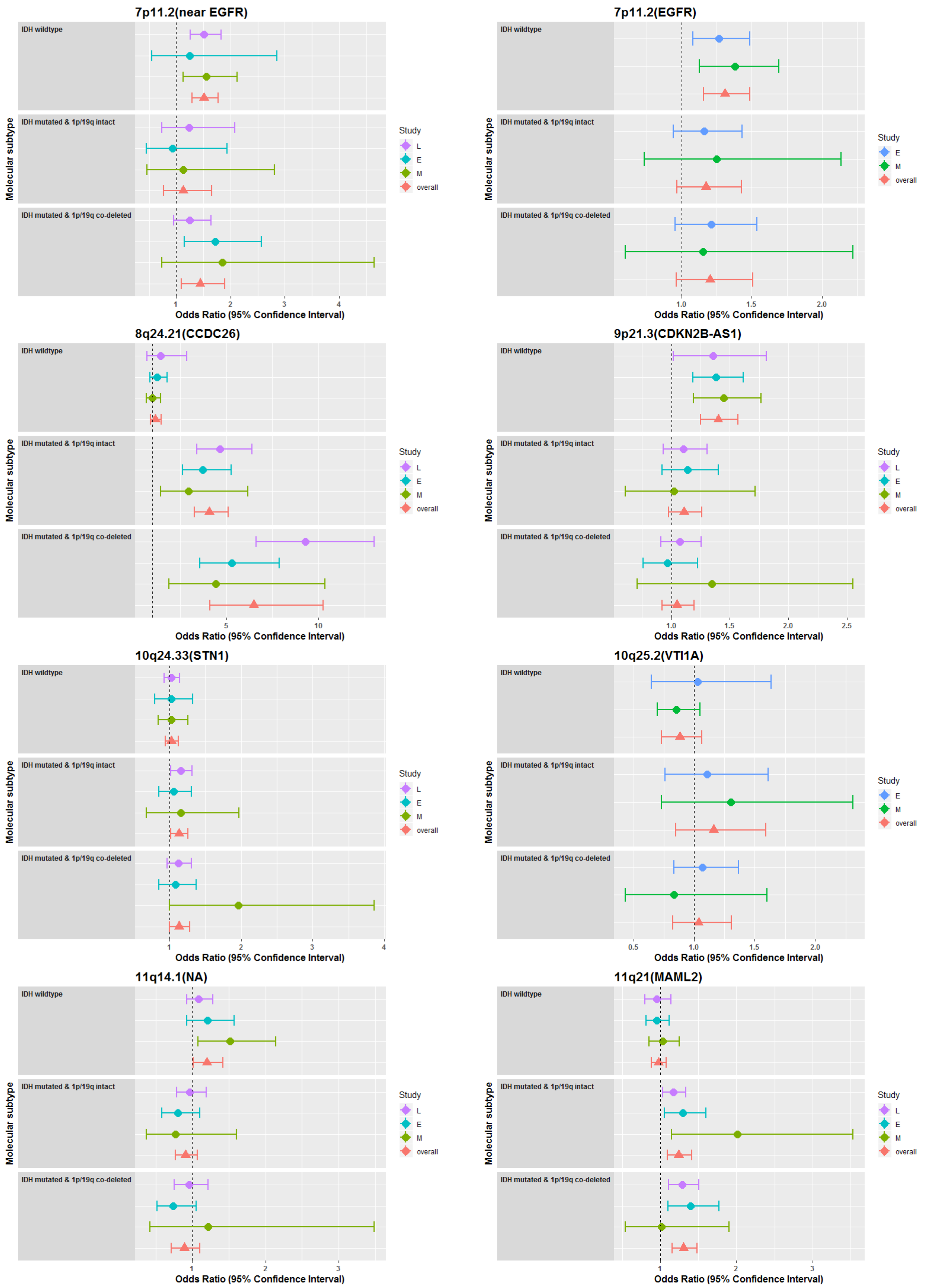
7p11.2(near EGFR)
7p11.2(EGFR)
8q24.21(CCDC26)
9p21.3(CDKN2B-AS1)
10q24.33(STN1)
10q25.2(VTI1A)
11q14.1(NA)
11q21(MAML2)
IDH wildtype
IDH mutated & 1p/19q intact
IDH mutated & 1p/19q co-deleted
Molecular subtype
Odds Ratio (95% Confidence Interval)
Study
L
E
M
overall

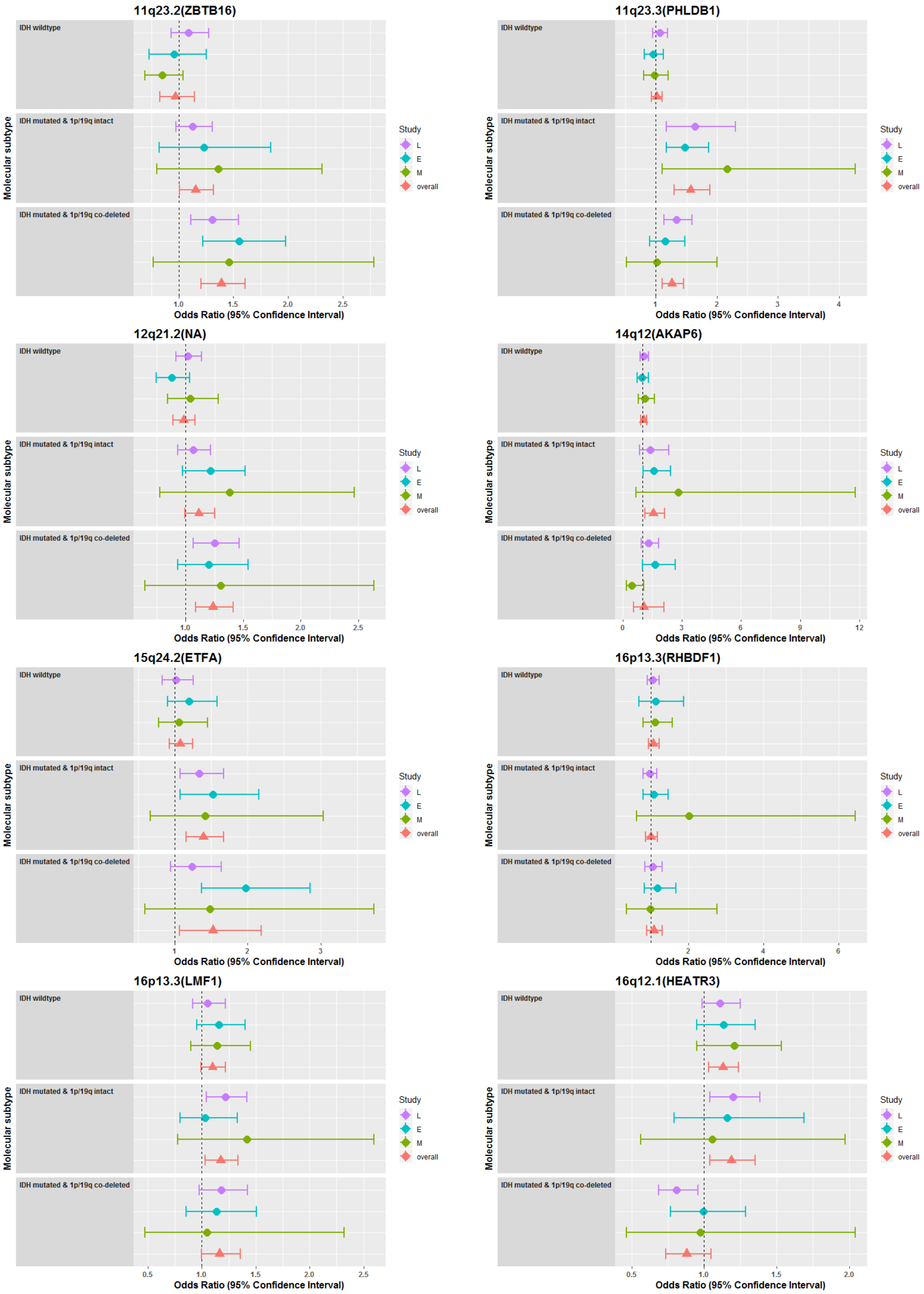
11q23.2(ZBTB16)
11q23.3(PHLDB1)
12q21.2(NA)
14q12(AKAP6)
15q24.2(ETFA)
16p13.3(RHBDF1)
16p13.3(LMF1)
16q12.1(HEATR3)
IDH wildtype
IDH mutated & 1p/19q intact
IDH mutated & 1p/19q co-deleted
Molecular subtype
Odds Ratio (95% Confidence Interval)
Study
L
E
M
overall

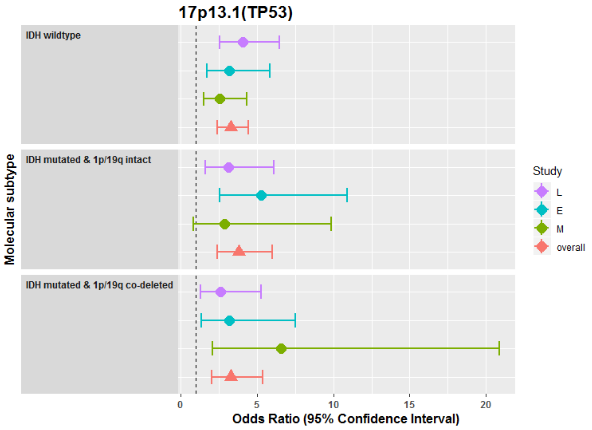
17p13.1(TP53)
Molecular subtype
IDH wildtype
IDH mutated & 1p/19q intact
IDH mutated & 1p/19q co-deleted
0
5
10
15
20
Odds Ratio (95% Confidence Interval)
Study
L
E
M
overall

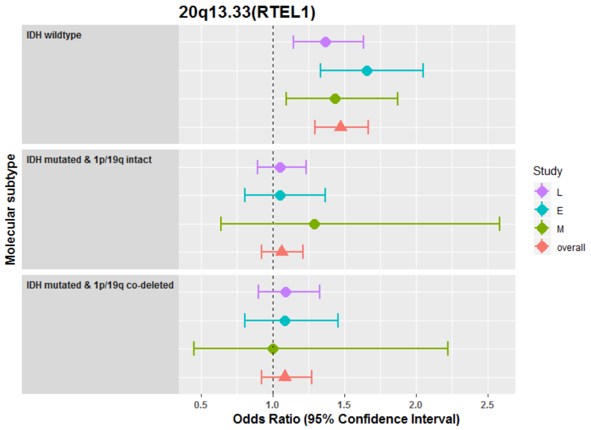
20q13.33(RTEL1)
Molecular subtype
IDH wildtype
IDH mutated & 1p/19q intact
IDH mutated & 1p/19q co-deleted
0.5
1.0
1.5
2.0
2.5
Odds Ratio (95% Confidence Interval)
Study
L
E
M
overall

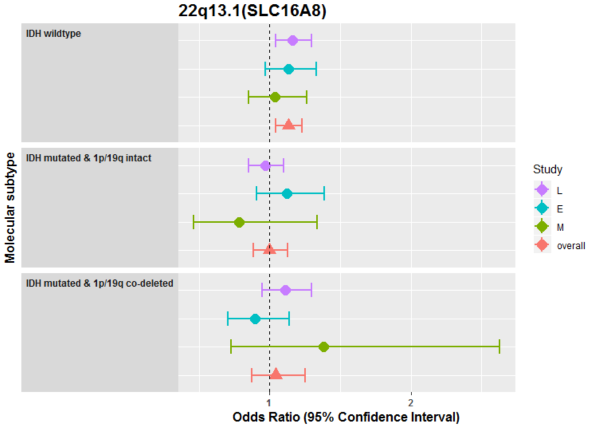
22q13.1(SLC16A8)
Molecular subtype
IDH wildtype
IDH mutated & 1p/19q intact
IDH mutated & 1p/19q co-deleted
1
2
Odds Ratio (95% Confidence Interval)
Study
L
E
M
overall

Figure S2. Representative images showing IDH1-R132H positive/mutated (a) and negative/wildtype staining (b).

(a) IDH1-R132H Positive/mutated

(b) IDH1-R132H Negative/wildtype

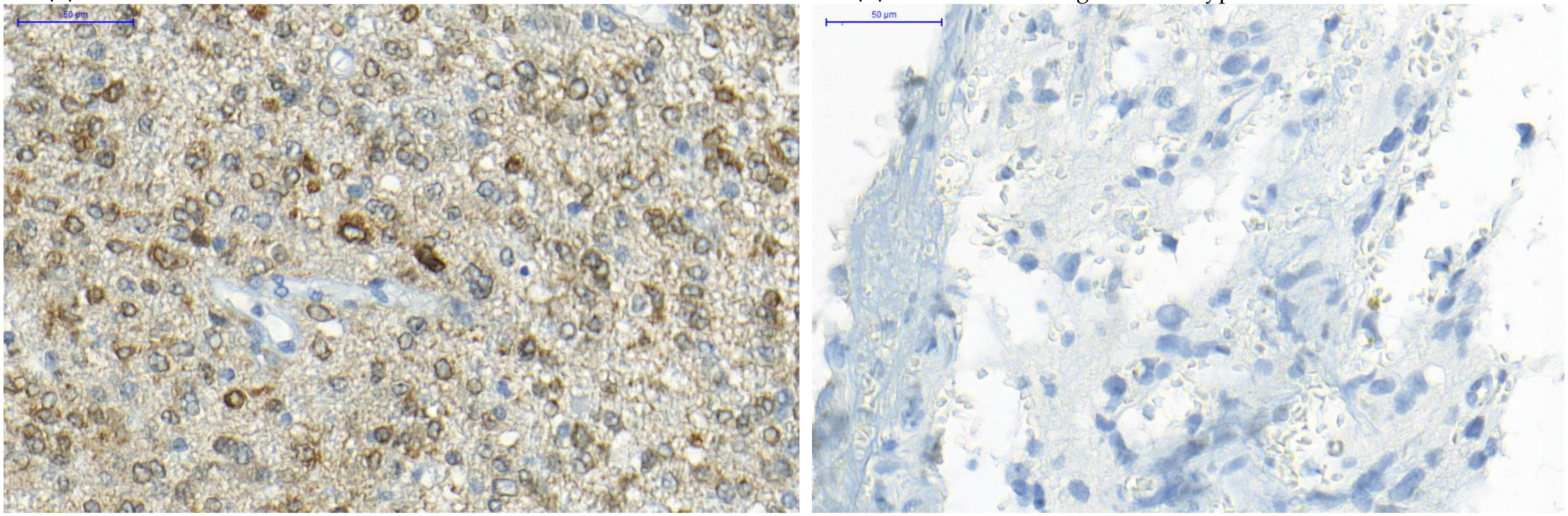

Figure S3. Representative staining showing the quality of our 1p/19q co-deletion assay. The 1p and 19q probes are red, while the control probes located on 1q and 19p are in green. The glioma on the left side is polyploid for both 1p and 19q, with no sign of deletion. The glioma on the right side has clear deletion of both 1p and 19q.

No deletion

1p/19q co-deletion

1p

19
q

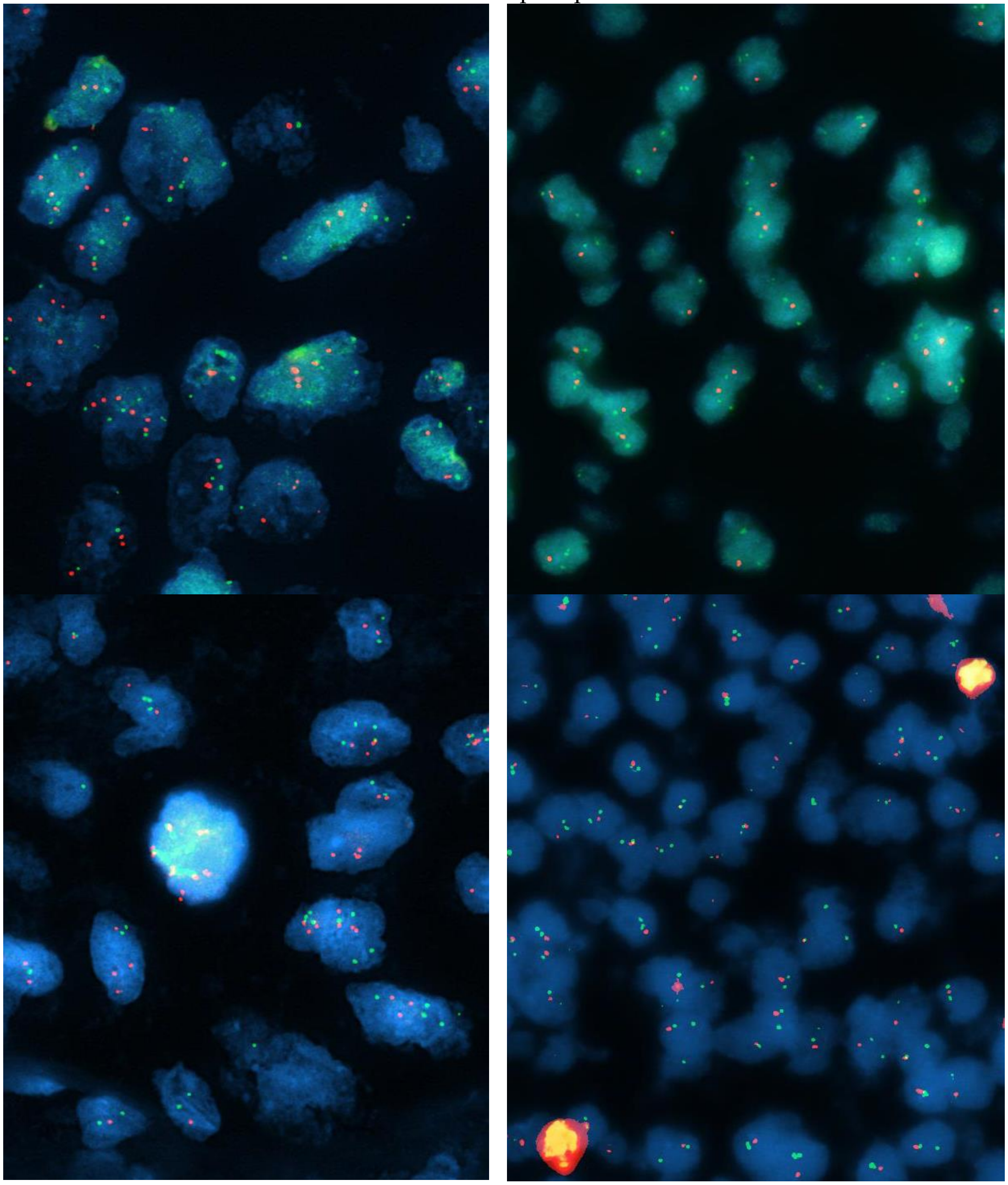

Supplement: Supplementary file 1 [file cancers-11-02001-s001.pdf]
